# The use of PDSA methodology to evaluate and optimise an inner city memory clinic: a quality improvement project

**DOI:** 10.1186/1471-2318-14-4

**Published:** 2014-01-15

**Authors:** Jennifer Perry, Francesca Bell, Therese Shaw, Barbara Fitzpatrick, Elizabeth L Sampson

**Affiliations:** 1 START Team (Assertive Outreach Team for Homeless People in Southwark, Lambeth and Lewisham), South London and Maudsley NHS Trust, London, UK; 2 Specialist CAMHS, Milton Keynes Community Health Service, Central and North West London NHS Trust, Milton Keynes, UK; 3 Community Old Age Psychiatry, St Ann’s Hospital, Barnet Enfield and Haringey Mental Health Trust, London, UK; 4 Haringey Memory Service, St Ann’s Hospital, Barnet Enfield and Haringey Mental Health Trust, London, UK; 5 Marie Curie Palliative Care Research Unit, UCL Mental Health Sciences Unit, University College London Medical School, London, UK

**Keywords:** Memory service, Dementia, Quality improvement

## Abstract

The Memory Services National Accreditation Programme states that memory services should provide “timely access to assessment and diagnosis” of dementia. We undertook a quality improvement project using Plan-Do-Study-Act methodology to improve patient access to an inner city memory service. This report focuses on the third Plan-Do-Study-Act cycle where, in 2012, we aimed to shorten the time from memory service referral to assessment and to diagnosis. The time from referral to assessment increased but the time from referral to diagnosis and to treatment decreased. Other memory clinics could use Plan-Do-Study-Act to enable faster diagnosis and better care for patients with dementia.

## Background

With the aging population and people living longer, the number of people with dementia is projected to increase significantly [1]. The “Improving Services for People with Dementia” report [2] highlighted that early diagnosis of dementia and intervention is cost effective. The Memory Services National Accreditation Programme (MSNAP) works with services to help them improve their quality of care. MSNAP guidelines state that a memory service should provide “timely access to assessment and diagnosis” [3]. In 2012 the Prime Minister, David Cameron, in his Dementia Challenge supported the Royal College of Psychiatrists in their work to increase the proportion of accredited memory services. The Haringey Memory Service (HMS), which this report focuses on, achieved accreditation in 2013.

The HMS provides care to people living in the London borough of Haringey. With a total population of 254,900, it is the fourth most deprived London borough and is ethnically diverse. 34.7% of the population are “White British” and of the remaining 65.3%; “Other White” comprise 23.0%, “Black African” 9.0%, and “Black Caribbean” 7.1% [4,5].

The HMS is based at St Ann’s Hospital, Haringey, and is staffed by psychiatrists, specialist nurses and mental health workers. It is part of Barnet, Enfield and Haringey Mental Health Trust which provides a range of services (including community mental health teams (CMHTs), home treatment and inpatient services) for people with dementia across the three boroughs. The service takes referrals from GPs for people aged over 65 with memory concerns. It offers specialist skills in assessment, diagnosis and treatment of dementia.

We developed a quality improvement program to reduce patient waiting times in the HMS using Plan-Do-Study-Act (PDSA) methodology [6].

## Method

PDSA is a tool for *“developing, testing and implementing changes leading to improvement”* which has four stages [6]. A plan is developed to test the change (Plan) and the test is carried out (Do). The data from the test is examined and reflected on (Study). Changes are planned or implemented for the next cycle of change (Act).

We examined a sample of 20 patients who were referred to the HMS in 2007. The mean waiting time from referral to assessment was 35.7 weeks; this was used as our baseline data. From 2007 onwards more consultant sessions and nurse led clinics were implemented.

The aim for the first PDSA cycle (2008/9) was that the time from referral to assessment decrease compared with the 2007 data (Plan). A sample of 20 patients referred in 2008/9 was examined (Do) and the mean waiting time was calculated as 29.7 weeks which showed a decrease (Study). Further consultant sessions and nurse led clinics were implemented (Act).

The aim for the second PDSA cycle (2011) was that the time from referral to assessment decrease compared with 2008/9 following the interventions (Plan). A sample of patients referred in 2011 was examined (Do) and the mean waiting time was calculated as 9.3 weeks which showed a decrease (Study). Interventions were made which included; liaising with the intake team (central referral team), GPs, CMHTs and radiology to reduce delays (Act).

**Figure 1:**
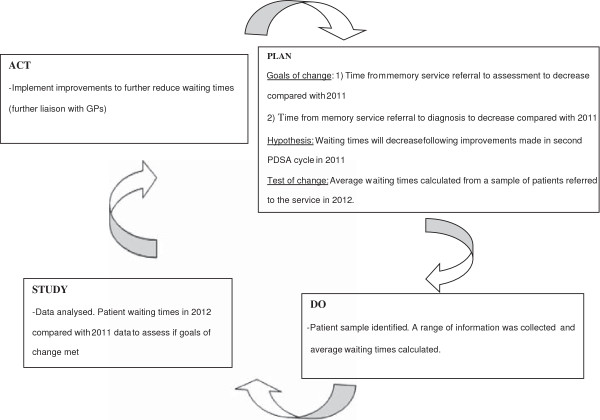
Memory Service Third PDSA Cycle (2012).

This report focuses on the third PDSA cycle (2012) (Figure 1). In the “Plan” phase the first aim was that the time taken from memory service referral to assessment should be shorter compared with 2011. The second aim was that the time taken from referral to diagnosis also be shorter compared with 2011. We wanted to demonstrate that there had been an improvement in the service through decreased patient waiting times as a result of the 2011 interventions.

In the “Do” phase a range of information was gathered on all patients referred to the HMS (21/4/12-11/10/12). The HMS referral book and patients’ medical records were used. This covered the same period used in the second PDSA cycle (21/4/11-11/10/11).

## Results

In the “Study” phase the results were analysed. 156 patients were referred to the HMS during this period. Forty cases were excluded at the first stage (referral to initial assessment) for a variety of reasons including; patients who did not attend their first appointment and patients with pending appointments.

Thirty seven patients were excluded at the second stage (initial assessment to diagnosis) for a variety of reasons including; patients who already had a diagnosis and those who had their diagnoses pending.

There were more referrals to the HMS during the 2012 period (156) compared with the same period in 2011 (102). After stage 1 exclusions this reduced to 116 referrals (2012) vs 100 referrals (2011) (Table 1). After stage 2 this reduced to 79 referrals both in 2011 and 2012.

**Table 1:** Memory Service Referral Information

	**Total number of referrals**	**Proportion of GP referrals**	**Proportion of CMHT referrals**	**Proportion of ‘Other’ referrals**
2012	116	70/116 (60%)	41/116 (35%)	5/116 (4%)
2011	100	66/100 (66%)	24/100 (24%)	10/100 (10%)

### Source of referral

60% (70/116) of referrals came from GPs, 35% (41/116) came from the CMHT and 4% (5/116) came from other sources (Drug and alcohol services, out of borough CMHTs/hospital).

### Demographics

The mean age of patients was 81.6 years.

The largest proportion of referrals was for patients who were “White British” (45%), followed by “White Other” (20%) and then by “Black/Black British” (17%). The proportion of other ethnicities referred was 2-6%.

### Pathway

The time taken from referral to initial assessment appointment increased compared with 2011 (mean: 10.9 vs 9.3 weeks, median: 9.9 vs 8 weeks, range: 2.6-30.6 vs 1.9-28.7 weeks).

**Figure 2:**
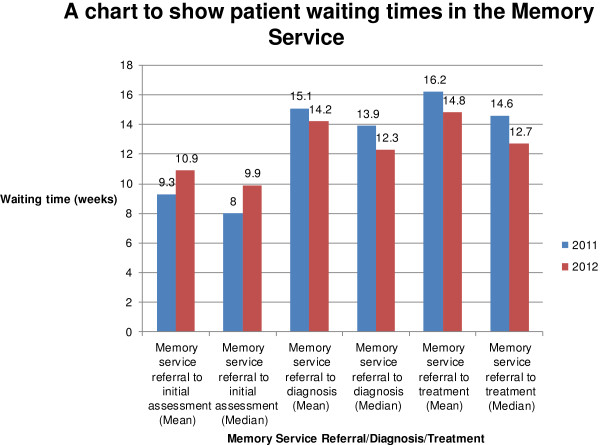
Graph to show patient waiting times in the memory service.

The time taken from referral to diagnosis decreased compared with 2011 (mean: 14.2 vs 15.1 weeks, median: 12.3 vs 13.9 weeks, range: 2.6-33 vs 4–71.1 weeks) as did time from referral to treatment (mean: 14.8 vs 16.2 weeks, median: 12.7 vs 14.6 weeks, range: 17–162 vs 22–498 weeks). 40/79 patients were started on treatment in 2012 (vs 39/79 in 2011) (Figure 2).

### Delays in Pathway

Potential delays in the pathway were examined. The time taken for GP referrals to be processed by intake decreased compared with 2011 (mean: 1.3 vs 1.8 weeks, median: 0.9 vs 1.4 weeks, range: 0.4-22.7 vs 0–11.1 weeks).

32 patients had a brain scan following their initial assessment. The median length of time taken to request the scan and obtain the results decreased compared with 2011 (median: 4.4 vs 4.9 weeks, range: 2–20.2 vs 9–50 weeks).

The delay between CMHT referral (for patients with memory difficulties as their presenting problem) and initial HMS assessment decreased compared with 2011 (mean 28.2 vs 39.2 weeks, median: 20.7 vs 47.1 weeks, range: 8.6-72.3 vs 8.9-131.9 weeks).

**Figure 3:**
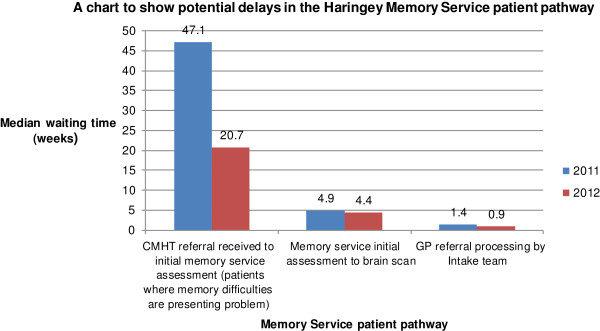
Graph to show delays in the Haringey Memory Service patient pathway.

The proportion of GP referrals with an incomplete past medical history and drug history increased compared with 2011 (31% vs 26%). The proportion of GP referrals with incomplete or no bloods also increased (70% in 2012 vs 59% in 2011) (Figure 3).

## Discussion

The largest proportion of referrals came from “White British” patients suggesting that this group may have different access to services in Haringey compared with other ethnic groups.

There was an increase in the number of referrals in 2012 which was most likely due to increased public awareness, the government’s dementia campaign and the promotion of the HMS to GPs. The increased number of referrals reflects the current national situation and has likely accounted for the increased waiting time from memory service referral to assessment.

The time taken from referral to diagnosis and from referral to treatment has decreased compared with 2011. This suggests that the improvements made in the second PDSA cycle have streamlined the pathway.

Delays involving brain scans being obtained and referrals being processed by the intake team and CMHT have all decreased compared with 2011. This shows that the interventions of liaising with the intake team, the CMHT and radiology department have been successful.

The proportion of GP referrals with an incomplete past medical history and drug history, or with incomplete or no bloods, has increased compared with 2011. The reasons for this finding are unclear as there was an intervention where letters were sent out to GPs promoting the HMS and reminding them of the referral requirements. These incomplete referrals may be leading to avoidable delays.

In summary, a number of mechanisms have driven change in this memory service. There have been a number of improvements made internally by the HMS and a number of extrinsic drivers such as the government campaign and increased media coverage of dementia. PDSA [6] is a well-recognised quality improvement methodology which has enabled us to evaluate and improve this memory service.

## Conclusions

The second aim of this third PDSA cycle has been met, however the first aim has not. Further issues need to be addressed in order to make the service pathway more efficient. In the “Act” phase of this PDSA cycle further liaison work will be undertaken with GPs so that referrals contain all the necessary information. Given the projected increase in the number of people developing dementia [1], service provision will need to expand in order to accommodate increasing referral numbers.

Consideration should be given as to how to better promote the memory service to Non White-British ethnic groups in Haringey given that they were under-represented in the clinic.

This quality improvement process and the MSNAP programme will be ongoing to ensure that the HMS pathway is further streamlined and improved. PDSA methodology [6] could be used in other memory clinics to reduce waiting times in the diagnosis and treatment of dementia.

## Abbreviations

CMHT: Community mental health team; HMS: Haringey memory service; MSNAP: Memory services national accreditation programme; PDSA: Plan-Do-Study- Act.

## Competing interests

The authors declare that they have no competing interests.

## Authors’ contributions

JP- Contributed to design of project, data collection and analysis. Drafted manuscript. Read and approved final manuscript. FB- Contributed to design of project, data collection and analysis. Drafted manuscript. Read and approved final manuscript. BF- Contributed to conception and design of project. Drafted manuscript. Read and approved final manuscript. TS- Contributed to conception and design of project. Drafted manuscript. Read and approved final manuscript. ES- Contributed to conception and design of project. Drafted manuscript. Read and approved final manuscript.

## Pre-publication history

The pre-publication history for this paper can be accessed here:

http://www.biomedcentral.com/1471-2318/14/4/prepub
